# Spermatocytic Variant of Classic Seminoma: A Report of Five Cases and a Brief Review of the Literature

**DOI:** 10.5041/RMMJ.10155

**Published:** 2014-07-25

**Authors:** Moshe E. Stein, Tomer Charas, Karen Drumea, Edmund Sabo, Rahamim Ben-Yosef

**Affiliations:** 1 Northern Israel Oncology Center, Rambam Health Care Campus, Haifa, Israel;; 2 Pathology Institute, Rambam Health Care Campus, Haifa, Israel and; 3 Faculty of Medicine, Technion-Israel Institute of Technology, Haifa, Israel

**Keywords:** Excellent prognosis, radiation therapy, spermatocytic seminoma

## Abstract

**Background:**

Spermatocytic seminoma is a rare testicular malignancy, appearing in the adult population. It has a good prognosis and a low rate of metastatic potential.

**Objectives:**

We present five cases diagnosed and treated with radiotherapy at Rambam Health Care Campus in Haifa, Israel.

**Methods:**

Between 1974 and 1996, five patients with stage I spermatocytic seminoma were referred post-orchiectomy to the Northern Israel Oncology Center. All five patients presented with the typical pathological features of the spermatocytic variant of classic seminoma, and all were staged clinically and radiologically.

**Results:**

Mean age at diagnosis was 44 years (range 30–58 years). Main symptoms included a palpable testicular mass and/or testicular enlargement. Mean duration of symptoms was 9 months (range 0.5–24 months). Three patients were irradiated to the para-aortic/ipsilateral iliacal lymph nodes (mean total dose 2,500 cGy), one patient with 4,000 cGy. One patient was irradiated to the bilateral iliacal lymph nodes (2,600 cGy). With a median follow-up of 15 years, four patients are alive with no evidence of disease or severe late side effects. One patient developed severe lymphedema and symptomatic peripheral vascular disease, stage IIA prostate carcinoma (hormonal and brachytherapy treatment) and a non-secretory hypophyseal adenoma (surgically removed); he died at the age of 75 due to severe peripheral vascular and coronary heart disease with no evidence of his first or second primaries.

**Conclusions:**

Prognosis is excellent and does not differ from classic seminoma. As in the accumulated experience in early-stage, low-risk classic seminoma, we suggest surveillance as the preferred policy.

## INTRODUCTION

The spermatocytic variant (SV) of classic seminoma (CS) is a rare testicular malignancy, first identified as a distinct tumor type and thoroughly described by Masson in 1946.^1^ It occurs mainly in men aged 50 years and older (average 53.5 years) and represents between 1% and 2% of all seminomas and 0.61% of all testicular germ cell tumors.^2^ Histogenetically, spermatocytic variant of classic seminoma (SVCS) cells belong to pre-meiotic germ cells (spermatocytes and spermatogonia). The spermatocytic variant is distinct from CS in its morphological characteristics with three different cell types (small, medium, large), spherical nuclei, eosinophilic to amphophilic cytoplasm, lack of cytoplasmic glycogen, and sparse to absent lymphocytic infiltrate (Table 1).^3^ Unlike CS, it occurs solely in the testis, is not associated with intra-epithelial testicular neoplasia, and presents bilaterally with a higher frequency than CS (10% versus 2%–4%). The tumor has a low propensity to metastasize, although metastatic SVCS, aggressive anaplastic variants of SVCS, and SVCS associated with sarcoma have been described.^2^,^3^ Generally, the SVCS is radiosensitive like CS, and radiation therapy at the same dose and volume as in CS can be used, although surveillance policy is preferred in low-risk seminoma. We describe our experience and long-term follow-up with five cases of SVCS staged and treated with radiation therapy between 1974 and 1996.

**Table 1.** Clinical and Pathological Comparison of Spermatocytic Variant of Classic Seminoma (SVCS) with Classic Seminoma (CS).

	**Spermatocytic Seminoma**	**Classic Seminoma**
**CLINICAL**		
1. Site of origin	Testis only	Testis, ovary, retroperitoneum, central nervous system (midline structures)
2. Arise in cryptorchid testes	No	10%
3. Age (years): mean (range)	54 (25–87)	41 (childhood to 85+)
4. Fraction of testis involved by tumor	2%	40%
5. Associated other germ cell tumor types	None	Common
6. Association with sarcoma of testis	5%	None
**MICROSCOPIC PATHOLOGY**		
1. Cell size	Small, medium, large	Medium
2. Nuclei	Spherical	Irregular
3. Cytoplasm	Eosinophilic to amphophilic	Pale to clear
4. Cytoplasmic glycogen	Absent	Abundant
5. Edema fluid	Often present	Absent
6. Lymphocytic infiltrate	Sparse to absent	Prominent
7. Syncytiotrophoblast	None	Occasional
8. Granulomas	None	Occasional
9. Lymphocyte-rich fibrovascular septae	Absent	Present
10. Associates intratubular germ cell tumor	None	Common
11. Microcystic pseudoglandular formation	Present	Absent
**MOLECULAR BIOLOGY/IMMUNOHISTOCHEMISTRY**		
1. Placental alkaline phosphatase staining	Rarely	Strong, diffuse
2. CD-117 staining	Absent	Present
3. Cytokeratin 18	Absent	Present
4. S-phase fraction	Twice as great as classic seminoma	
5. DNA content	<3N	>3N
6. Gene overexpression chromosome 9	Positive	Negative

## PATIENTS AND METHODS

Between 1971 and 2010, 162 post-orchiectomy seminoma patients were referred to the Northern Israel Oncology Center for staging and treatment (112 had stage I disease). Five stage I patients, diagnosed between 1974 and 1996, demonstrated the pathological features of SVCS described by Eble.^3^ Patients were staged biochemically with the specific tumor markers (beta-human chorionic gonadotrophin, B-HCG; alpha-fetoprotein, AFP; and serum lactic dehydrogenase, LDH), chest radiography, and whole-body computerized tomography (CT) scan, while two patients underwent lymphangiography which was abandoned in the 1990s. Testicular ultrasound was performed prior to the surgical procedure.

Since 1990, radiotherapy is delivered to all stage I seminoma patients using megavoltage (MV) photons to a total dose of 2,500 cGy in 200-cGy daily fractions, prescribed to the midplane as described by Warde et al.^4^ Follow-up was measured from the date of diagnosis (date of orchiectomy) until last follow-up.

## RESULTS

Mean age of patients at diagnosis was 44 years (range 30–58 years). Two were Jewish, and three were Arabic. Only two patients were born in Israel. The right testicle was the site of the tumor in three patients. All were pT1/stage I disease, and the tumor was confined to the testis with no extension beyond in all patients. No patient had a history of maldescended testis or gonadal dysgenesis. Main symptoms included painless testicular mass or enlargement and a mean duration of symptoms of 9 months (range 0.5–24 months). Systemic symptoms, such as fever, loss of weight or appetite, and severe pain, were not reported.

Three patients were treated with the “hockey stick” method with radiation therapy to the paraaortic and ipsilateral iliacal lymph nodes, and one patient was irradiated to the bilateral iliacal lymph nodes (“inverted-Y”), all with a mean total dose of 2,562 cGy (range 2500–2600 cGy) and daily fractions of 200 cGy. One patient was treated to a total dose of 4,000 cGy in the “hockey stick” manner. Treatment facilities included linear accelerators (6–18 MV) and one Cobalt-60 (Co-60) machine.

Due to risk factors for local spread, such as herniorraphy and scrotal biopsy, the inguinal/scar areas were boosted in three patients to a median dose of 2,200 cGy (range 1,560–2,600 cGy) with mean daily fractions of 220 cGy.

After a median follow-up of 15 years, four patients are alive with no evidence of recurrent disease, second primary, or severe radiation-induced late side effects. One patient (aged 39 years) underwent a scrotal biopsy prior to his left inguinal orchiectomy which demonstrated pathological spermatocytic seminoma with typical and atypical seminoma. He was treated with the “hockey stick” method (para-aortic and left hemi-pelvis) to a total dose of 4,000 cGy (daily fractions of 200 cGy) and was boosted with Co-60 to the left inguinal region to a dose of 1,560 cGy (daily fractions of 200 cGy). Side effects were severe lymphedema and vascular disease of the left leg. Approximately 26 years after completion of his scheduled radiotherapy program, prostate carcinoma (stage IIA) was diagnosed and successfully treated with brachytherapy and hormonal therapy. Three years later, a nonsecretory hypophyseal adenoma was diagnosed and removed. This patient died at the age of 75 (36 years after his orchiectomy), due to severe peripheral vascular and coronary heart disease with no evidence of his malignancies.

## DISCUSSION

The spermatocytic variant of classic seminoma (SVCS) has been regarded as a malignancy along the lines of CS, but it exhibits different pathology and natural history, albeit the same clinical behavior. It is an uncommon tumor and, at our institution, represents less than 1% of all CS patients and 4.4% of stage I CS. The usual age at diagnosis SVCS is over 50 years, but between 30 and 40 years for CS;^5^ in our series, however, one patient presented at the age of 30 years. SVCS has never been documented in the pre-pubertal age, although recent data indicate a much wider age distribution than previously reported (from 19 to 92 years).^6^

Regarding etiology, maldescent of the testis does not appear to be a significant predisposing factor for SVCS, except for a single reported case in a 44-year-old man.^6^ Several studies have reported a slight prevalence in the right testis.^2^ As in our study, most patients experienced a painlessly enlarging testicular mass, and the mass had been recognized by the patient for 12 months or longer in about one-third of cases and for 5 years in one case.^3^,^7^ One of our patients consulted his physician only after 12 months of symptomless testicular enlargement.

Serum markers, such as B-HCG and AFP, as in our study, when reported, have been invariably negative. The clinical, microscopic-morphologic, and immunohistochemical features of SVCS and CS are clearly depicted in Table 1.

To date, about 240 cases of SPS have been reported.^2^,^3^ The overwhelming majority of cases presented with stage I disease, and only a few cases have been described with metastatic disease.^8^ The common management described by those patients parallels that of CS, radiation therapy to the para-aortic and ipsilateral iliacal lymph nodes with omission of the scar and the inguinal region, to a dose amounting to 2,000–2,500 cGy with excellent long-term survival.^9^ Due to the rarity of this tumor type and missing (or failing) long-term survival data, there are only sporadic data about radiation-induced second primaries.

Currently, the management of stage I seminoma has changed to the increased use of surveillance, provided that there are no risk factors which may predict recurrence.^10^ Predictive factors for relapse (rete testis invasion, 4 cm or greater size of original primary tumor) have been described in multivariable analysis by Warde et al.^11^ The risk of a contralateral tumor appears to exceed that of meta-static disease, 10% in SVCS, whereas the corresponding figure for CS appears to be 2%–4%.^3^,^12^ A regular testicular ultrasound should be an integral part of the diagnostic work-up, along with surveillance policy and physical examination, blood count, biochemistry profile including the specific tumor markers, and CT scan.

About 16 cases of SVCS that underwent sarcomatous transformation have been described so far.^2^,^3^,^13^ In most, the sarcomatous component had rhabdomyosarcomatous features, and in other cases the sarcomatous component was of undifferentiated spindle cell type and even elements of chondrosarcoma.^3^,^14^ The development of sarcoma occurs either by differentiation of totipotential germ cells to somatic tissues and subsequent malignant transformation, or by malignant transformation of preexisting teratomatous elements.^15^ The sarcomatous elements were admixed within the tumor, but the metastatic disease developed from the sarcomatous parts with a very poor prognosis and most patients died of metastatic disease a few months after diagnosis.

In the anaplastic variant of the SVCS, there was an anaplastic component of the medium-sized cells, comprising up to 10%–40% of the tumor mass, against a background of conventional spermatocytic features of the seminoma. Other unique pathological features were a multinodular growth pattern with nodules of various sizes separated by fibrous septa; tumor giant cells with high degree of atypia; moderately high mitotic rate with the presence of atypical forms; area of necrosis and vascular invasion; and a Ki-67 index of 30%–40% in areas with anaplastic features.^2^,^16^ No more than six cases of the anaplastic variant have been described to date.^2^,^16^,^17^ These patients presented with a painless, rapidly growing mass or testicular enlargement. All underwent orchiectomy, three were treated with additional radiotherapy, two with two cycles of cisplatinum-based chemotherapy, and all are reported to be alive with no evidence of recurrent disease. The presence of the anaplastic component does not seem to impact the excellent prognosis.

## CONCLUSION

In conclusion, SV of CS is associated with a favorable outcome. Due to the excellent prognosis, the policy of post-orchiectomy surveillance in low-risk stage I disease has been adopted. The presence of anaplastic components within the SV does not have any negative impact on survival or recurrence rate.

## Abbreviations:

AFP: alpha-fetoprotein;
B-HCG: beta-chorionic gonadotrophin;
Co-60: Cobalt-60; CS, classic seminoma;
CT: computerized tomography;
LDH: lactic dehydrogenase;
MV: megavoltage;
SVCS: spermatocytic variant of classic seminoma.
